# COLLABORATIVE PARTNERSHIP BETWEEN STATE AGENCIES AND ACADEMIA ADDRESSING LONG-TERM CARE WORKFORCE NEEDS

**DOI:** 10.1093/geroni/igac059.1966

**Published:** 2022-12-20

**Authors:** Katie Ehlman, Karla Diekemper, Kevin Valadares

**Affiliations:** University of Southern Indiana, Evansville, Indiana, United States; University of Southern Indiana, Evansville, Indiana, United States; University of Southern Indiana, Evansville, Indiana, United States

## Abstract

The rural areas of southwestern Indiana are challenged with insufficient healthcare workers in the long-term care industry. The University of Southern Indiana (USI) Geriatric Workforce Enhancement Program (GWEP) is collaborating with state agencies to address shortages in licensed administrators, activity professionals and social service designees. These roles, vital in the nursing facility, hold educational requirements through the Indiana State Department of Health (ISDH) and the Indiana Professional Licensing Agency (IPLA). USI gerontology faculty addressed workforce challenges in five phases: 1) Evaluation of state educational requirement and comparison to current USI gerontology courses; 2) Development of new coursework to support state requirements; 3) Expansion of preceptor pool; 4) Proposal to state agencies; 5) USI curriculum modifications. Faculty evaluated the educational and training requirements for these roles and compared the requirements with current courses. Preceptors were needed for students’ field experiences; a preceptor pool was developed through funded training. The requirements and course alignment were included in the program proposal to the state agencies. Upon ISDH, IPLA and University New Program Development Committee approval, additional instructors were recruited. Results of this initiative include a: 1) Fully approved and launched Administrator-in-Training (AIT) residency embedded in curriculum at undergraduate and graduate level; 2) Fully approved Activity Professional Certificate Program; 3) Pool of 10 certified preceptors; and 4) Onset of the social service designee certificate program. This collaborative partnership developed between USI, ISDH and IPLA ensure state agency requirements are met academically for students entering the long-term care workforce.

